# Notes and Notices

**Published:** 1896-03

**Authors:** 


### NOTES AND NOTICES.

DR. A. T. NOE, formerly of Kirksville, Mo., has removed to 2911 Lafayette Avenue, St. Louis, Mo.

DR. HOMER I. OSTROM announces to the profession that his "Private Surgical Hospital," 127 West Forty-seventh Street, New York, is completed and that he is now prepared to receive surgical cases and those requiring surgical operations. Telephone 936, Thirty-eighth Street.

DR. W. A. YINGLING has removed to 814 Market Street, Emporia, Kansas.

DR. C. S. FAHNESTOCK, the eminent surgeon, is the Dean of Dunham Medical College. Dr. Fahnestock for many years practiced medicine at Laporte, Ind.

STIBIUM ARSENICOSUM is an excellent remedy in chronic bronchitis. It is reported to have cured a case of six years' duration, where there was great emaciation, the pulse 115, the temperature 103, and severe night-sweats and incessant cough added to the seriousness of the condition. Its special characteristics are great rattling in the chest, restlessness, and scant expectoration.—*Med. Century*, March, 1896.

PLUMBUM METALLICUM.—Constant constipation with headache.

DR. CHARLES F. HITCHCOCK has removed from Buffalo, New York, to Sodus, New York, where he succeeds to the practice of Dr. Whittleton.

INDIANA INSTITUTE OF HOMŒOPATHY.—The thirtieth annual meeting of the Indiana Institute of Homœopathy will convene in the parlors of the Grand Hotel, Indianapolis, Wednesday and Thursday, May 20th and 21st, 1896. Come; let us compare notes, and, by so doing, take a step forward in the "Art of Healing." Bring a non-member with you. Railroad rates will be secured. Rates will be given at the Grand Hotel. The busy doctors write the papers. Will you report a case from your practice, or write a practical paper to read at this meeting? Send at once to the Secretary the title of your paper, so the final programme can be completed. Officers: President, E. Z. Cole, M. D.,

Michigan City; Vice-President, G. O. Erni, M. D., New Albany; Treasurer, J. S. Martin, M. D., Muncie; Secretary, F. C. Stewart, M. D., Indianapolis. Censors: W. R. Elder, M. D., Terre Haute; J. A. Compton, M. D., Indianapolis; A. H. Sears, M. D., Anderson; F. H. Huron, M. D., Danville; H. M. Logee, M. D., Connersville. Publication Committee: W. R. Stewart, M. D., Indianapolis; I. D. George, M. D., Indianapolis; R. W. Rodgers, M. D., Indianapolis.

Dr. Mary A. Cooke has removed from 2107 North Seventeenth Street to 2113 North Eighteenth Street, Philadelphia, Pa.

The Medical Century respectfully announces that it has established a New York Department, in charge of Dr. J. Perry Seward, No. 113 West Eighty-fifth Street, who is authorized to represent it as reportorial and business correspondent for New York and vicinity. Courtesies extended Dr. Seward in this relation will be highly appreciated at the home office.

C. E. Fisher, M. D., *Editor.*

The Standard Dictionary.—A very vicious attack on the Funk & Wagnalls *Standard Dictionary* appeared some time since in the Minneapolis *Tribune*, seemingly with editorial authority. It now appears that a rival publisher was responsible for the attack.

The following editorial note is conspicuously printed in the Minneapolis *Tribune:*

"In certain advertisements heretofore published in this paper, certain statements reflecting upon the *Standard Dictionary*, published by Funk & Wagnalls Company, of New York, have been made. Lest the impression should be had that the *Tribune* originated these statements, and has given them circulation on its own account, we wish to say the *Tribune* was not and is not responsible for these statements, and that the *Tribune does not indorse the charges therein contained.* These charges were made by the purchasers of those advertisements."

Nervousness of Motormen.—The following statement of the nervous condition of men who are employed to operate some of the modern systems of transportation in the cities will be read with interest by all medical men:

Neurologists are watching with a considerable degree of interest a new expression of a nervous malady which has made itself manifest since the introduction of the Broadway cable cars and the Brooklyn trolley system. With the exception of Chicago, there are no other cities possessing anything like the street traffic of New York and where these methods of transportation are in operation. A nervous condition, not at all like the usual nervousness that is excited by great noise, confusion, or sudden danger, has developed itself in several gripmen employed on the Broadway road, and among the motormen employed on the Brooklyn trolley lines.

The constant lookout for collisions in the congested district below Canal Street in Broadway keeps the gripman in a state of extreme nervous tension from the time he goes on his car until he goes off. Besides keeping an eye open for visible trouble, his mind is fixed on possibilities that are under his feet. He doesn't know just when there is to be a pooling of interests between the grip and a broken strand in the cable, which will whisk him along the street, crashing into trucks, smashing wagons, frightening pedestrians, and exasperating policemen. This continuous strain results, first, in sleeplessness, then in a falling off in appetite, and extreme irritability; after this a tremor in the facial muscles. At the expiration of a week these symptoms disappear, and may not return for ten days, but thereafter the intervals are regular and are about one week apart—seven days in a state of nervous terror and seven days in a normal, apparently healthy condition. These exhibitions apply only to men of nervous, nervo-sanguine, and bilious temperaments. While present in other temperaments, they are not pronounced.—*Medical Examiner.*

IN the advertisement in the February number of Theodore Metcalf Co., of Boston, there will be found an offer that is exceedingly liberal, and one our readers should avail themselves of. It is not often that $15 is exchanged for $5.

"PLEASURES OF OUTDOOR LIFE."—Live while you live. Get all legitimate pleasure you can. This is a beautiful world. Don't miss a large part of its pleasure by going through life blindfolded, as many people do. The out-door world is poetic, pleasing, instructive. There's a wealth of pleasure in roaming over the hills, across the fields, or through the woods. All nature is in harmony of music to the attentive ear. Birds, plants, flowers, ferns, mosses, insects, the beauty of minerals, yes, even the stars above, are strains in this harmony. Get in closer touch. Take *The Observer*, Portland, Conn. Sample, 10 cents. One year, $1.

QUININE KILLS A CHILD.—Martha Hudock, aged seven months, of Freeland, Pa., died January 25th, 1896, from quinine poisoning. A servant gave the child a box of pills to play with. The lid became detached and the child ate the contents, some thirty grains of quinine. Dr. Wright, who was hastily summoned, was unable to overcome the effects of the poison with emetics, and the sufferer died in great agony.—*The Philadelphia Times.*

CHILDREN'S HOMŒOPATHIC HOSPITAL—THE ANNUAL ELECTION OF THE OFFICERS AND MANAGERS OF THE INSTITUTION.—At the annual meeting of the Children's Homœopathic Hospital, Broad Street, above Poplar, the following Directors were appointed: To serve for three years, Bushrod W. James, M. D.; Edward H. Binns, Samuel R. Marshall, Lewis P. Posey, M. D., and Albert B. Weiner; to serve for two years, Landreth W. Thompson, M. D.; M. M. Walker, M. D.; Joseph M. Reeves, M. D.; Napoleon B. Kelly, and Daniel A. Waters; to serve for one year, William J. Johnson, Silas Griffith, M. D.; N. F. Lane, M. D.; Thomas M. Longcope, and Captain Henry L. Gregg. Solicitor, Henry R. Edmunds. At the meeting of the Board of Directors the following officers were elected for the year: President, Dr. Bushrod W. James; Vice-President, Samuel R. Marshall; Treasurer, Edward H. Binns; Secretary, Napoleon B. Kelly; Associate, Dr. N. F. Lane.

